# Novel traits of *Trichoderma* predicted through the analysis of its secretome

**DOI:** 10.1111/j.1574-6968.2012.02665.x

**Published:** 2012-09-18

**Authors:** Irina S Druzhinina, Ekaterina Shelest, Christian P Kubicek

**Affiliations:** 1 Research Division Biotechnology and Microbiology, Institute of Chemical Engineering, Vienna University of TechnologyVienna, Austria; 2 Austrian Center of Industrial Biotechnology (ACIB), GmBH c/o Institute of Chemical Engineering, Vienna University of TechnologyVienna, Austria; 3 Systems Biology/Bioinformatics Research Group, Leibniz Institute for Natural Product Research and Infection Biology, Hans Knoell InstituteJena, Germany

**Keywords:** mycoparasitism, environmental opportunism, chitinases, cellulases, small secreted cysteine-rich proteins, proteases

## Abstract

Mycotrophic species of *Trichoderma* are among the most common fungi isolated from free soil, dead wood and as parasites on sporocarps of other fungi (mycoparasites). In addition, they undergo various other biotrophic associations ranging from rhizosphere colonization and endophytism up to facultative pathogenesis on such animals as roundworms and humans. Together with occurrence on a variety of less common substrata (marine invertebrates, artificial materials, indoor habitats), these lifestyles illustrate a wealthy opportunistic potential of the fungus. One tropical species, *Trichoderma reesei*, has become a prominent producer of cellulases and hemicellulases, whereas several other species are applied in agriculture for the biological control of phytopathogenic fungi. The sequencing of the complete genomes of the three species (*T. reesei, T. virens,* and *T. atroviride*) has led to a deepened understanding of *Trichoderma* lifestyle and its molecular physiology. In this review, we present the *in silico* predicted secretome of *Trichoderma*, and – in addition to the unique features of carbohydrate active enzymes – demonstrate the importance of such protein families as proteases, oxidative enzymes, and small cysteine-rich proteins, all of that received little attention in *Trichoderma* genetics so far. We also discuss the link between *Trichoderma* secretome and biology of the fungus.

## Introduction

*Trichoderma*^1^ (teleomorph *Hypocrea*, Ascomycota, Dikarya) species are among the most common fungi frequently isolated as saprotrophs from free soil, soil litter, dead wood and rhizhosphere. Most of *Trichoderma* species are mycotrophs as they grow on the mature sporophores of other fungi (necrotrophic mycoparasitism), yet some (e.g., *H. jecorina*/*T. reesei*) are also found on decaying wood, often in close proximity to other fungi (Druzhinina *et al*., 2011). In addition, they are found on a panopticon of other substrata, including xenobiotically infested soil, kerosene tanks in aircrafts, and indoor building walls (Klein *et al*., 1998). Finally, *Trichoderma* spp. undergo various biotrophic associations ranging from rhizosphere colonization and endophytism up to facultative pathogenesis on such animals as roundworms and humans (Druzhinina *et al*., 2011). This multitude of ecological niches illustrates the successful strategies for environmental opportunism and competition for habitats.

Two of these properties have brought *Trichoderma* into a broad interest: first, a single available wild-type strain of *T. reesei* has become the progenitor of a multitude of mutants that are contemporarily used in biotechnological industry for the production of cellulases and hemicellulases that are applied for food and feed, textile, and particularly for biofuel production (Kubicek, 2012a). Second, the ability to antagonize, parasitize on or even kill other fungi has initially been the reason for exploitation of some species (especially *T. harzianum* sensu lato, *T. atroviride (H. atroviridis), T. virens (H. virens), T. asperellum,* and *T. asperelloides*) for the biological control (biocontrol) of phytopathogenic fungi in the field (Harman *et al*., 2004). The more recent discovery that some of these fungi can also establish themselves in rhizosphere, stimulate plant growth, and elicit plant defense reactions led to an additional boost in research on the application of *Trichoderma* as biofertilizer (Harman, 2011).

All the above-mentioned ecological traits of *Trichoderma* require, among others, the secretion of proteins for breaking down the polymeric organic molecules into a form that can be absorbed. In addition, they also secrete proteins that can act as toxins or signals for communication with mutualistic partners. It is clear that the vital property of any opportunist including *Trichoderma* is a successful combination of a superior ability to degrade multiple polymers and to associate (in a broad sense) with other (micro)organisms. Therefore, the inventory of secretome of an organism may reveal its potential ecological adaptations.

Recently, the genomes of the three *Trichoderma* species (*T. reesei*, *T. virens,* and *T. atroviride*) have been sequenced by DOE JGI (Joint Genome Institute, http://genome.jgi.doe.gov, Grigoriev *et al*., 2011) and annotated (Martinez *et al*., 2008; Kubicek *et al*., 2011). In this review, we describe the composition and properties of the *in silico* predicted secretome of *Trichoderma* and explain how the respective findings add to our understanding of the molecular physiology and ecology of the fungus.

## The *Trichoderma* secretome

The secretome of an organism can be predicted from its protein sequences by *in silico* tools (e.g., SignalP Petersen *et al*., 2011; http://www.cbs.dtu.dk/services/SignalP/) that screen for a signal sequence at the N-terminus used to move it into the endoplasmic reticulum for post-translational processing and ultimate secretion. Lists of such *Trichoderma* proteins can be retrieved from the Sordariomycetes page of the MycoCosm portal of DOE JGI (http://genome.jgi.doe.gov/sordariomycetes/sordariomycetes.info.html). When the probability of the signal peptide presence is setup for 95% (*P* < 0.05), secretomes of *T. reesei, T. atroviride,* and *T. virens* are defined by 826, 1030, and 1096 putative proteins, respectively. However, not all of them are truly excreted into the medium, but may stay in the endoplasmic reticulum or are transferred to vacuole or plasma membranes. Therefore, we applied the TMHMM Server v2.0 (http://www.cbs.dtu.dk/services/TMHMM/) to predict transmembrane helices in proteins, ProtComp, v8.0 (http://linux1.softberry.com/) and WolfPsort (http://wolfpsort.org/) both designed to predict the subcellular localization for animal or fungal proteins to remove these proteins. So, 747, 968, and 947 proteins of *T. reesei, T. atroviride,* and *T. virens*, respectively, were predicted as secreted through the plasma membrane (Table 1). Enzymes acting on poly- and oligosaccharides (carbohydrate active enzymes or CAZymes), small secreted cysteine-rich proteins (SSCPs), and ‘unknown proteins’ (i.e., orthologs for which in other ascomycetes have no function identified yet) comprised > 60% of the secretome of all three species (as determined in July 2012). Enzymes with hydrolytic activity on other polymers (proteases, lipases, nucleases, and phosphatases) and orphan proteins (i.e., proteins that have either no orthologs or only in other *Trichoderma* species) were present in lower numbers (as for July 2012). Interestingly, a small number of proteins were identified as enzymes that require molecular oxygen as substrate (flavoprotein monooxygenases, copper radical oxidases, cytochrome P450 oxidoreductases) and enzymes acting on hydrogen peroxide and superoxide (Table 1).

**Table 1:** Composition of the predicted secretome of *Trichoderma*

	*T. reesei*	*T. atroviride*	*T. virens*
Total genes	9143	11 865	12 518
Genes with signal peptide	826	1030	1096
Extracellular proteins	747	968	947
Glycosyl hydrolases (GH)	114	140	139
Proteases	61	81	63
Oxidases	24	32	28
SSCPs	174	258	250
Unknown genes	213	264	253
Orphan genes	50	78	63
Other genes	111	115	151

Oligonucleotide array data are so far available only for *T. reesei*, but a comparison of the predicted secretome with the genes expressed under a variety of conditions such as growth on cellulose and lactose or in presence/absence of light (Ivanova *et al*., 2012; Arvas *et al*., 2011; Tisch *et al*., 2012) reveals that 560 of the 672 genes that encode secretory proteins are indeed expressed. The nontranscribed 17% of genes of *T. reesei* are evenly distributed among all the groups described earlier (Table 1). Notably, proteomic studies related to cellulase and hemicellulase formation in *T. reesei* have so far described a much smaller number of secreted proteins (cf. Herpoël-Gimbert *et al*., 2009; Jun *et al*., 2011; Adav *et al*., 2011; for review see Kubicek, 2012a). In the most comprehensive analysis to date (Adav *et al*., 2011), 50 cellulases, hemicellulases, and cellulase-accessory proteins, respectively, were identified, which make up less than half of the 122 CAZymes that are predicted to be secreted. It is possible that many of these proteins are secreted under *in vitro* (but maybe also *in vivo*) only in low amounts so that they remain undetected by experimental methods, or that many of them are proteins that are either part of the cell wall or remain bound to it.

Adav *et al*. (2011) also detected the presence of several of the proteins that perform an oxidative attack on extracellular substrates (cytochrome P450 monooxygenases, FAD-dependent enzymes etc.).

Two proteomic studies (Jun *et al*., 2011; Adav *et al*., 2011) also detected several enzymes of intermediary metabolism (such as dehydrogenases) in the culture filtrate. While their presence could be due to autolysis or mycelial fragmentation (Kubicek, 2012a), several of such enzymes were also predicted as secreted. The biological role of their secretion, if it indeed occurs, remains unknown.

### Degrading fungal cell wall polysaccharides

**Fig 1:**
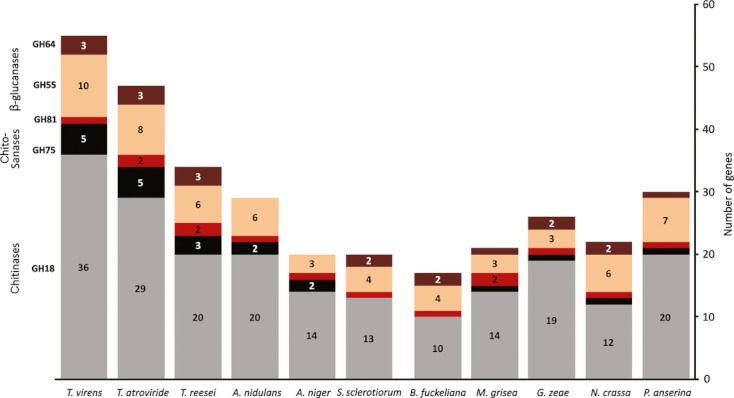
Amplification of chitinases, chitosanases, and ß-glucanases in *Trichoderma*. The numbers indicate genes. Generic names for fungi are as follows: *T*. for *Trichoderma, A*. for *Aspergillus, S*. for *Sclerotinia, B*. for *Botryotinia*, *M*. for *Magnaporthe, G*. for *Gibberella, N*. for *Neurospora,* and *P*. for *Podospora,* respectively.

*Trichoderma* does not degrade lignin; thus, the majority of the polymers that it may target in its environment are polysaccharides. As a tribute to this, glycosyl hydrolases (GH) make up for about 15% of the *Trichoderma* secretome and comprise 122 of the 200 predicted CAZymes (Martinez *et al*., 2008). While similar trend is also seen in other fungi, a comparative analysis reveals the abundance of genes encoding chitin- and ß-glucan degrading enzymes, in which *Trichoderma* (particularly the vigorous mycoparasites *T. virens* and *T. atroviride*) outnumber other fungi (Fig. 1). A reason for this may be deduced when one considers that the structural scaffold of the fungal cell wall is composed of chitin – a β-(1,4)-linked *N*-acetylglucosamine polymer – and β-(1,3)-glucan, which are together embedded in the amorphous fraction of α-glucans, galactomannans, and other carbohydrate polymers (Latgé, 2007). The enhancement of the gene inventory for the degradation of these polymers is therefore in accordance with the mycotrophic nature of *Trichoderma*. This is also reflected in *T. reesei,* which is a weaker mycoparasite compared to *T. atroviride* or *T. virens*, although it is capable to attack other fungi (Druzhinina *et al*., 2010). The chitinases have recently been described in detail by Seidl (2008) and Gruber & Seidl-Seiboth (2012). Besides chitin, the second fibrillar polymer in fungal cell walls is β-(1,3)-glucan (Latgé, 2007). β-(1,3)-glucanases have so far been described for GH families 16, 55, 64, and 81. An increased numbers of GH enzymes from families 55 and 64 were also found in *T. atroviride* and *T. virens* compared to other filamentous fungi (Kubicek *et al*., 2011). Because some of the β-(1,3)-glucan also contains β-(1,6) branches, β-(1,6)-glucanases are also present. Their deduction from GH families is more difficult; however, they are found in GH5 and GH30, but members of both of these families have different activities ranging from mannosidases to cellulases and xylosidases (for details, see Carbohydrate-Active enZYmes Database at http://www.cazy.org).

While an important role of chitinases and ß-glucanases appears logical in terms of the necessity of *Trichoderma* to break up the wall of other fungi in mycotrophy, it is remarkable that the corresponding genes are not induced during confrontation with *Rhizoctonia solani (Thanatephorus* spp.). Only the GH16 endo-ß-1,3/1,4-glucanases of *T*. *atroviride* is significantly upregulated during this process (L. Atanasova, C.P. Kubicek, I.S. Druzhinina, unpublished).

### Proteases

Our analysis reveals that *Trichoderma* may have one of the largest sets of proteases among fungi (as predicted with use of the peptidase database MEROPS http://merops.sanger.ac.uk, Rawlings *et al*., 2012). Indeed, the total numbers of predicted proteases are 383 (4.2% of all predicted protein coding genes), 445 (3.75%), and 479 (3.85%) in *T. reesei*, *T. atroviride*, and *T. virens*, respectively. Roughly, around 20% of the deduced *Trichoderma* proteases possess a signal peptide and are therefore entering the secretory pathway. The dominant groups were aspartyl proteases, serine proteases, subtilisin-like proteases, dipeptidyl and tripeptidyl peptidases (Table 2). The number of representatives of most of the secreted protease families is nearly the same between the species, but several families, such as the subtilisin-like proteases of the S8 family, dipeptidyl (S9) and tripeptidyl peptidases (S33), are expanded in *T. virens* and *T. atroviride* compared to *T. reesei* (Kubicek *et al*., 2011). An interesting exception, however, was the alkaline serine proteases that are present in *T. atroviride,* of which only one had orthologs in *T. reesei* and *T. virens*.

**Table 2:** Proteolytic enzymes in the *Trichoderma* secretome*

Active amino acid residue	Number	Family	*A. fumigatus*	*T. reesei*	*T. atroviride*	*T. virens*
Aspartate	A1	Aspartyl proteases	7	17 (15)	19 (16)	17 (15)
Cysteine	C01A	Papain type			1	
C13	Legumin family			1	1
Glutamate	G1	Aspergillopepsin	3	1	5	1
Metal ion	M01	Aminopeptidase N				1
M12	ADAM family of metalloproteases	2		1	1
M14	Metallocarboxypeptidase	1	4	4	4
M18	Aminopeptidase 1 type	1	1	1	1
M20A	Glutamatecarboxypeptidase type	3	4	3	4
M24	Methionyl aminopeptidase 1	3	7	7	6
M28	Aminopeptidase S	5	7	7	7
M35	Deuterolysin	2	1	1	1
M36	Fungalysin	1	1	1	1
M38	Isoaspartyl dipeptidase			1	
M43	Cytophagalysin family	1		1	
Serine	S01A	Chymotrypsin		1	1	2
S8	Subtilisin	2	11 (10)	15 (13)	15 (13)
S9B	Dipeptidyl peptidase	4	12 (9)	14 (10)	21 (15)
S10	Carboxypeptidase Y	11	3	4	3
S12	D-Ala-D-Ala-carboxypeptidase B		2	1	3
S33	Prolyl aminopeptidase		5 (4)	9 (5)	10 (7)
S41	Serine endopeptidase	1			
S51	Dipeptidase E		1		1
S53	Sedolisin	6	3	11	7
Threonine	T03	γ-glutamyl transferase		1	1	2

* Where two numbers are given, the first number is based on intact MEROPS output and the number in brackets specifies those that remained after manual removal of doubtful predictions.

It is also striking that, despite the rather similar total number of protease families in the three *Trichoderma* spp., about 75% of them did not contain orthologs in the other two species and were thus unique. This phenomenon was most strongly reflected in the dipeptidyl peptidases, which shared no orthologs at all, and the least expressed in the aminopeptidases, which consisted of 80% orthologs.

A comparison of secreted proteases of *Trichoderma* with those of dermatophytic fungi (Burmester *et al*., 2011) shows the similarity in the enlargement of four M14 metallopeptidases and the S8 subtilisin family. In addition, the S33 serine proteases displayed in the *Trichoderma* secretome are mostly not secreted in other fungi.

This unique pattern of expanded and secreted protease families likely offers an advantage to opportunistic *Trichoderma* species. Thus, recent transcriptomic studies (Seidl *et al*., 2009) showed that the attack of *R. solani* (as a model prey) by *T. reesei, T. atroviride,* and *T. virens* was accompanied by the expression of various families of extracellular proteases and transporters for oligopeptides and amino acids. While some proteases from *Trichoderma* (mainly *T. harzianum*) had been implicated previously as involved in mycoparasitism (for review see Benítez *et al*., 2004), these mostly comprised aspartyl proteases, subtilisin-like proteases, and the alkaline protease Prl1 (the latter only in *T. atroviride*; see above). In contrast, transcriptomic analysis identified – besides the two mentioned groups – also the involvement of dipeptidyl- and tripeptidyl peptidases and metalloproteases (L Atanasova, CP Kubicek, IS Druzhinina, unpublished). We speculate that the diversity of these proteases may form a synergistic system for the efficient use of proteins by *Trichoderma*.

This amplified arsenal of proteases may not only be needed for the attack of other fungi, but also be involved in the interaction with nematodes and human tissues by respective species (*T. longibrachiatum* and its close genetic neighbors). Sharon *et al*. (2001) demonstrated improved nematode antagonism in a *T.* cf. *harzianum* strain, in which the alkaline protease gene PLR1 has been overexpressed. A ‘serine protease’ from *T. pseudokoningii* SMF2 was recently shown to have nematocidal activity killing juveniles and inhibiting egg hatching of the plant parasitic nematode *Meloidogyne incognita* (Chen *et al*., 2009). Similarly, proteases are the key virulence determinants produced by entomopathogenic fungi *Metarhizium anisopliae* and *Beauveria bassiana* (*Cordyceps bassiana*) during pathogenesis, and strains overexpressing protease Pr1A are being developed as alternatives to chemical insecticides (Schrank & Vainstein, 2010). In addition, various proteases have been implicated in virulence by fungi pathogenic to warm-blooded animals including humans such as *Aspergillus fumigatus* (*Neosartorya fumigate*), *Candida albicans,* or *Microsporum canis*, although the results are still controversial (Monod *et al*., 2002; Monod, 2008; Burmester *et al*., 2011). In the amphibian pathogenic chytrid, *Batrachochytrium dendrobatidis*, significant lineage-specific expansions in three protease families (metallo-, serine-type, and aspartyl proteases) were found to have occurred during transition from its nonpathogenic ancestor (Joneson *et al*., 2011).

### Degradation of plant biomass

*Trichoderma reesei* QM 6a, a fungus originally isolated as ‘*Trichoderma viride*’ during World War II at Guadalcanal on the Solomon Islands, is the progenitor of a plethora of high cellulases producing mutant strains. Nevertheless, the leading position of *T*. *reesei* in cellulase production has recently been questioned by the findings that its genome contains only a small repertoire of genes encoding cellulases and hemicellulases (Martinez *et al*., 2008) when compared to several other fungi. Yet, a more detailed comparison with inclusion of the two other *Trichoderma* spp. shows that this generalization is only partially true (Table 3): the number of different cellulase components including the class 1 cellulose binding domains (CBM1) in *Trichoderma* is in the same range as found in other Pezizomycotina. *Trichoderma* spp., however, have much less GH61 polysaccharide monooxygenases. They dramatically increase cellulose hydrolysis by cellulase mixtures in a metal-ion-dependent way (Harris *et al*., 2010). Phillips *et al*. (2011) showed that the *N. crassa* polysaccharide monooxygenases display different regiospecificities on the cellulose chain resulting in oxidized products modified at either the reducing or nonreducing end of the glucan chain. In *N*. *crassa*, the electron transfer necessary for their action comes from cellobiose dehydrogenase. This is interesting because *Trichoderma* – while showing the enhancement of cellulase hydrolysis by the addition of the GH61 proteins (Harris *et al*., 2010) has no obvious ortholog of cellobiose dehydrogenase (C.P. Kubicek, unpublished data). Yet, *T*. *reesei* culture filtrates can oxidize cellulose (Szakmary *et al*., 1991), and it must therefore possess alternative systems for electron transfer, identification of which is clearly a point for further studies.

**Table 3:** Members of CAZymes families containing cellulases, cellulose monooxygenase, and cellulose binding proteins in *Trichoderma* spp. and other Pezizomycotina*

	GH1	GH3	GH5	GH6	GH7	GH12	GH45	GH61	CBM1	Total
*Trichoderma reesei*	2	13	11	1	2	2	1	3	15	50
*Trichoderma virens*	2	17	16	1	2	4	2	3	23	70
*Trichoderma atroviride*	4	14	14	1	2	3	1	3	19	61
*Gibberella zeae*	3	22	15	1	2	4	1	15	12	75
*Magnaporthe grisea*	2	19	13	3	6	3	1	17	22	86
*Neurospora crassa*	1	9	7	3	5	1	1	14	19	60
*Aspergillus nidulans*	3	21	16	2	3	1	1	9	7	63
*Aspergillus niger*	3	17	10	2	2	4	0	7	8	53
*Podospora anserina*	1	11	15	4	6	2	2	33	28	102
*Sclerotinia sclerotiorum*	3	13	14	1	3	5	2	9	19	69
*Botryotinia fuckeliana*	3	16	15	1	2	4	2	9	18	70

* Enzymes occurring in the listed GH families include the following: GH1, GH3: various ß-glycosidases, including ß-glucosidases, ß-mannosidases and ß-xylosidases; GH5: endo-ß-glycanases including endo-glucanases and endo-mannanases; GH6: cellobiohydrolase CEL6A; GH7: cellobiohydrolase, CEL7A and endo-ß-1,4-glucanase CEL7B; GH12, GH45: endo-ß-1,4-glucanases; GH61, cellulose monooxygenase; CBM1: cellulose binding domain type 1.

A comparison of the hemicellulase diversity also contradicts the generalization of the ‘poor enzyme inventory’: when again compared to other fungi, all three *Trichoderma* spp. are enriched in several hemicellulolytic components, such as GH27 α-galactosidases, GH54 α-arabinofuranosidases/ß-xylosidases, GH67 and GH79 α-methyl-glucuronidases, GH95 α-fucosidases, and GH30 glucuronyl-xylanases. However, it is also clear that *Trichoderma* is short in enzymes degrading arabinan and ß-mannan and most strikingly in enzymes degrading pectin: while all three *Trichoderma* spp. have GH28 pectin hydrolases and GH78 rhamnogalacturonases, they completely lack pectin, pectate, and rhamnogalacturonan hydrolases, and also – except for CE8 pectin methylesterases – lack all esterases acting on pectin. In most of these cases*, T. reesei* shows a tendency of having one or more genes less than *T. atroviride* and *T. virens*. This can be partially referred to the operation of repeat-induced point mutation (RIP), a gene silencing mechanism that occurs in sexually reproducing fungi (Selker, 1990; Kubicek *et al*., 2011).

What do these differences between *Trichoderma* and other fungi tell about the biology of the fungus? The inventory of cellulolytic and hemicellulolytic enzymes described earlier is consistent with an organism that does not directly attack lignocellulose, but rather feeds on the predigested cellulose and particular hemicelluloses (xylan, arabinan, galactan). The expanded GHs are particularly necessary to cleave bonds to side chain substituents within XXGG-type xyloglucans, rhamnogalacturonan I, cereal arabinoxylans, and hardwood glucuronoxylans – polymers that surround the cellulose in the plant cell wall (Kubicek, 2012b). Interestingly, we have recently observed that the tropical *T*. *reesei* enhances the induction of its whole cellulolytic and hemicellulolytic arsenal when facing a temperate *R*. *solani* that is a very unlikely prey/host for this species in nature, whereas such response is not observed with *T*. *atroviride* or *T*. *virens* (L. Atanasova, C.P. Kubicek, I.S. Druzhinina, unpublished). The presence of a basidiomycete fungus may thus signal the availability of predigested plant biomass to *T*. *reesei*, a finding fully in accordance with the hypothesis that this species became a saprotroph by following basidiomycetes into their habitat (Rossmann *et al*., 1999).

Yet, the *Trichoderma* secretome also contains a number of oxidative enzymes, whose function has not yet been investigated, but may contribute to plant biomass degradation. These include glucose oxidases, multicopper oxidases (including laccases), and copper radical oxidases (glycolate oxidases). The presence of these enzymes is typical for white rot fungi, where they participate in lignin degradation (Kersten & Cullen, 2007; Hammel & Cullen, 2008). However, in brown rot fungi such as *Postia placenta,* they aid in the degradation of cellulose by Fenton chemistry (Martinez *et al*., 2009). Could such a mechanism also operate in *Trichoderma*? One of the possible mechanisms working in brown rot fungi involves extracellular quinone redox cycling, quinate transporter, phenylalanine ammonia lyase, and laccase (Cohen *et al*., 2004). It is interesting that all these genes are in fact expressed in *T. reesei* in the presence of cellulose (R. Bischof, B. Seiboth & C.P. Kubicek, unpublished data). In addition, the elevated expression of iron-uptake system and ferrooxidoreductases may be necessary to avoid the formation of Fe^3+^-trioxalate chelates that are poorly reducible by hydroquinones (Jensen *et al*., 2001). The secretome of *Trichoderma* spp. also contains several oxaloacetate decarboxylases, of which one is induced on cellulose (R. Bischof, B. Seiboth & C.P. Kubicek, unpublished data). It can contribute to the detoxification of the wood predegraded by brown rot fungi that secrete oxalic acid into the medium (Micales & Highley, 1991).

### Detoxification

*Trichoderma* secretome also contains a considerable number of cytochromes P450 proteins. They represent a superfamily of sequence-related heme oxygenases, which are found in most organisms, and whose roles range from carbon-source degradation and the elaboration of metabolites in prokaryotes, lower eukaryotes and plants, to detoxification of xenobiotic compounds in insects and mammals including humans (Kelly *et al*., 2006; Singh *et al*., 2011). However, they play the major role in the extracellular inactivation of hazardous materials (Roelofs *et al*., 2012; Syed & Yadav, 2012). The detection of cytochrome P450 monooxygenases in *Trichoderma* secretome may explain the ability to grow and develop even in highly polluted habitats or in kerosene tanks (Klein *et al*., 1998). These enzymes may therefore be also important for *Trichoderma*'s opportunism.

### Small secreted cysteine-rich proteins (SSCPs)

Finally, one of the largest groups of proteins secreted by *Trichoderma* is the small secreted cysteine-rich proteins (SSCPs). They were identified by the criteria that their M_r_ should be ≤300 amino acids long (as recommended by Martin *et al*., 2008) and containing four or more cysteine residues (Kubicek *et al*., 2011). Sequence similarity search tools and phylogenetic analyses allow their further subdivision into four groups: (i) hydrophobins and hydrophobin-like proteins; (ii) elicitor-like proteins; (iii) proteins with similarity to MRSP1 (MAP kinase repressed secreted protein 1), a 16-kDa protein that was identified to be strongly overexpressed in a delta*-tmk1* (a MAPK) mutant of *T. virens* and which bears the conserved four-cysteine pattern C-X29-C[P/G]C-X31-C; and (iv) SSCPs with no attribution to any functional category (Kubicek *et al*., 2011).

Hydrophobins, probably the best known SSCPs, are characterized by the presence of eight positionally conserved cysteine residues of which four occur in doubles. They are found on the outer surfaces of cell walls of hyphae and conidia, where they mediate interactions between the fungus and the environment. Hydrophobins are conventionally grouped into two classes (classes I and II) according to their solubility in solvents, hydropathy profiles and spacing between the conserved cysteines. *Trichoderma* is known to have the largest abundance of class II hydrophobins (Kubicek *et al*., 2008). *Trichoderma atroviride* and *T. virens* – but not *T. reesei* – also have class I-like hydrophobins, which however deviate from the class I hydrophobins of other fungi in several aspects and form separate clades within ascomycete-wide phylogenetic analysis (Seidl-Seiboth *et al*., 2011). Hydrophobins are also involved in the attachment of *Trichoderma* hyphae to roots (Viterbo & Chet, 2006).

A second, emerging SSCP group, are the elicitor-like proteins. One of them, *T. virens* Sm1 (ortholog *T. atroviride* EPL1; Seidl *et al*., 2006) has also been studied in some detail, because it induces systemic disease resistance in the dicotyledon cotton (*Gossypium hirsutum*; Djonovic *et al*., 2007). Also, Sm1 is necessary for the induction of systemic protection of maize (*Zea mays*) via the jasmonic acid pathway (Djonovic *et al*., 2007). It is therefore likely that this group of SSCPs aids in the interaction of *Trichoderma* with plants. In fact, SSCPs with this action are known from several plant pathogenic fungi (Rep, 2005). However, in ectomycorrhizal basidiomycetes, SSCPs appear to be rather important in symbiotic interactions (Martin *et al*., 2008). One of the most strongly expressed SSCP genes of *Laccaria bicolor* (*miSSP7*) is necessary for the establishment of ectomycorrhizae (Plett *et al*., 2011; Plett & Martin, 2012).

The function of the proteins belonging to the largest and unique group of *Trichoderma* (iv) SSCPs cannot be predicted at present. Interestingly, however, some members of this cluster contain CFEM domains or consensus sequences for glycosylphosphatidylinositol (GPI anchors). Conservation of these genes suggests that they may encode cell surface proteins with important roles in the interaction with other organisms, as in *C. albicans* (Pérez *et al*., 2011).

## Concluding remarks

Although the availability of both genome sequences and bioinformatic tools makes the prediction of potentially secreted proteins relatively straightforward, it is interesting that this approach has so far only scarcely been used in fungal ecology. In this review, we have shown how such information can be used to detect new or overlooked physiological properties. The inventory of *Trichoderma* poly- and oligosaccharide hydrolytic and modifying enzymes was already well known. However, the richness of its proteolytic arsenal was shown for the first time. In addition, the presence of several types of oxidative enzymes (and their expression during growth on cellulose) offers the possibility of alternative modes for degradation of lignocelluloses. Probably, the most remarkable aspect is the high number of SSCPs and unknown and orphan proteins, which together accounted for about 50% of the secretome and even exceeded this value in the secretome expressed on cellulose. Such proteins could include key effectors that control recognition of macromolecules and/or recognition of *Trichoderma* partner organisms in native habitat. The fact that these proteins have hardly been described in proteomic analyses of *T*. *reesei* culture filtrates may be due to their small size, which may have led to their escape from the 2D gels. Given the fact that many enzyme preparations from *T*. *reesei* are manufactured with only little purification steps, it would be worthwhile to know about the action and properties of these proteins.

## Footnotes

1 In this review we accommodate the changes proposed at the International Botanical Congress in July 2011 for the International Code of Botanical Nomenclature. The ongoing discussion on the future single taxon name for *Hypocrea/Trichoderma* that may be followed at the website of the IUMS International Subcommission on *Trichoderma* taxonomy at http://www.isth.info/content.php?page_id=102. Therefore we use the single generic name *Trichoderma* not only for asexual species but also for holomorphs when the sexual stage is described. However at first mention of holomorphic species both teleomorph (*Hypocrea*) and anamorph (*Trichoderma*) names are given. When the whole genus is considered, the name *Trichoderma* is applied.
